# Knowledge of Animal Welfare and Consumers’ Behavioral Intentions in China: A Moderated Mediation Model of Product Cognition and Empathy

**DOI:** 10.3390/ani12081043

**Published:** 2022-04-16

**Authors:** Yaoming Liang, Gengrong Hua, Weiyou Cai, Gen Li, Hao Wang, Hui Li

**Affiliations:** 1 College of Economics & Management, South China Agricultural University, No. 483 Wushan Road, Tianhe District, Guangzhou 510642, China; evanliang@scau.edu.cn (Y.L.); hua_gr@foxmail.com (G.H.); 2 College of Veterinary Medicine, South China Agricultural University, Guangzhou 510642, China; ligen223@126.com; 3 Animal Disease Prevention and Control Center, Chenghai District, Shantou 515800, China; ccweiyou@163.com; 4 College of Public Administration, South China Agricultural University, Guangzhou 510642, China; 5 College of Natural Resources and Environment, South China Agricultural University, Guangzhou 510642, China

**Keywords:** animal welfare, knowledge, behavioral intention, product cognition, empathy

## Abstract

**Simple Summary:**

In this study, consumer perceptions of animal welfare have been assessed. The results can strongly support the development of policies and legislation regarding animal-friendly production. In China, the demand for animal-friendly products is increasing, but so far, the research on the relationship between the knowledge of animal welfare and animal-friendly consuming intentions is limited. The objective of this study was to examine the impact of the knowledge of animal welfare on consumers’ behavioral intentions and its mechanism. The survey covered 1499 food consumers in Guangdong province, China. Our empirical results suggest that increasing knowledge of animal welfare is significantly positive for the intention of animal-friendly products consumption. Furthermore, empathy moderates the indirect effect between animal-friendly product cognition and the behavioral intention both to purchase and recommend.

**Abstract:**

As purchase power and consumption knowledge increase, consumers gradually demand safer and healthier products. Animal welfare is expected to be an important attribute of high-end food in the future and a major concern for the high-quality development of the livestock industry. The objective was to shed new light on our understanding of consumers’ perceptions and behavioral intentions toward animal-friendly food. Using sample data of 1499 food consumers in Guangdong province, China, this study explored the role of product cognition and empathy in the relationship between consumers’ knowledge and behavioral intentions. Results indicate that knowledge of animal welfare significantly influences consumers’ behavioral intentions, and there is a mediating effect on cognition. Meanwhile, empathy moderates the relationship between product cognition and consumers’ intentions to purchase or recommend animal-friendly products. Improving consumers’ knowledge of animal welfare and cognitive levels of animal-friendly products may contribute to promoting animal-friendly product consumption and sustainable development of the livestock industry.

## 1. Introduction

**Figure 1:**
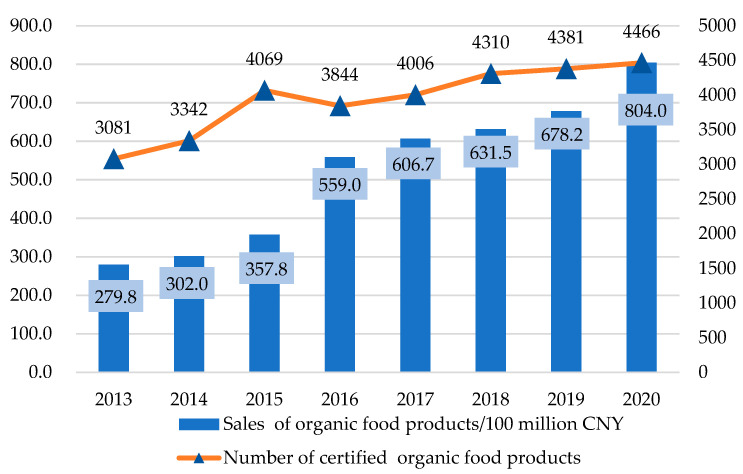
Trends of Chinese Organic Food Products. (Source: Green Food Development Center China and Prospective Industry Research Institute.)

Animal welfare has been a widely discussed topic in recent decades. It plays an important role in promoting food safety and quality and achieving sustainable development of animal husbandry. As the largest and most populous developing country worldwide, China has been making heroic efforts to address the issue of human welfare, yet animal welfare has been less of a concern. With the remarkable improvement in people’s living standards, Chinese people have paid more and more attention to a healthy diet and food production management processes. High-end foods represented by organic foods are increasingly popular with the public. The number of product varieties has risen roughly every year since the implementation of organic food acts (China’s Certification and Accreditation Administration (CNCA) developed Organic Product Certification Implementation Rules in 2005 and revised them in 2012, 2014, and 2019 to standardize the food certification process.) in 2012, and sales of organic food products have grown tremendously, reaching CNY 804 billion in 2020 (See Figure 1). The structure of public food consumption structure in China is moving toward an emphasis on quality together with economic development.

Animal welfare is generally regarded as one of the quality attributes of food products [1]. Although over 60% of respondents had never heard of the concept of animal welfare in mainland China [2], based on a survey in Jiangsu province, Wang and Gu [3] found that consumers were willing to pay 16.2% more for animal-friendly products when not informed about the correlation between animal welfare and meat quality, and 21.3% more when informed of that information. Though there is no animal welfare product label in China at present, consumers are increasingly turning to products with higher welfare standards in Chinese daily consumption, exhibiting a positive perception and increased demand. Additionally, a growing number of Chinese consumers seem to consume broiler chickens from the free-range poultry system, natural grain-fed fattening pigs, and milk products without exogenous agents such as antibiotics, etc. These products with animal welfare attributes also tend to be more expensive. For example, fresh tenderloin of ecological black pigs fed with Chinese herbal medicine for more than 300 days sold for 129 CNY/kg on JOYBUY (JOYBUY, powered by jd.com, is one of the largest e-commerce companies in China.) on 31 December 2020, and the price was at least 1.5 times higher than that for ordinary pork.

Animal welfare is consistent with ethical requirements. Numerous scientific studies have demonstrated that increasing animal welfare may benefit animal production and health and the quality of animal-derived products while minimizing food safety risks and other related issues in the livestock industry [4,5]. Chinese consumers’ willingness to pay (WTP) for animal welfare products may be because they think not only that animal welfare products are better, but also that animals deserve more consideration. In 2018, China promulgated the Law of Wildlife Protection, aiming to maintain biodiversity and ecological balance and promote the harmonious development of man and nature. Particularly, under the influence of the global pandemic caused by COVID-19 in 2020, China has comprehensively banned eating wild animals, and the concept of a "community of destiny" between humans and animals has become more popular. In 2021, China moved to introduce the Animal Epidemic Prevention Law to prevent, control, purify and eliminate animal epidemics, promote the development of aquaculture, prevent and control zoonotic infectious diseases, and ensure public health safety and human health. Farm animal welfare plays a crucial role in promoting food safety and quality and achieving the sustainable development goals of animal agriculture. Currently, Chinese consumers’ attitudes and willingness to pay for animal welfare improvements have been studied in a few articles [1,2,6]. There are also very few studies that show that information about the husbandry system may affect consumers’ willingness to pay for animal-friendly products [7]. However, research on the relationship between knowledge of animal welfare and consumers’ behavioral intentions in China remains rare.

As knowledge about animal welfare has increased, growing consumer demand has prompted the agricultural sector to adopt more sustainable and animal-friendly practices. The Chinese government is gradually becoming more aware of the need to provide higher standards of animal husbandry, so as to ensure farm animal health, the quality of animal-derived products, and the development of green agriculture. The International Cooperation Committee of Animal Welfare (ICCAW) of the China Association for the Promotion of International Agricultural Cooperation (CAPIAC) was approved by the Chinese Ministry of Agriculture in 2013. Since then, more regulations and policies have been introduced in China, such as Farm Animal Welfare Requirements: Pigs, China’s first set of farm animal welfare standards. Soon, a widening range of animal-friendly products will be available to meet the consumer demand in China. Furthermore, behavioral intentions are a strong predictor of behavioral performance and a prerequisite for behavior [8]. A more intensive understanding of Chinese consumers’ behavioral intentions regarding products with animal welfare attributes and in-depth research on its formation process and mechanism are urgently needed.

This paper is concerned with consumers’ behavioral intentions regarding animal welfare products in China, and in particular, examines the impact of animal welfare knowledge and product cognition on consumers’ behavioral intentions and its mechanism, and further seeks to investigate the role of empathy in the above mechanism. The main contributions of our work are reflected in the following aspects. Firstly, this study may shed new light on the relationship between knowledge of animal welfare and consumers’ behavioral intentions. Secondly, it may provide an analytical foundation for stakeholders to better understand the heterogeneity in Chinese consumers’ behavioral intentions toward animal welfare products and its intrinsic causes. And finally, the findings of this study can also provide a rationale for increasing demand for high-end animal husbandry products and promoting improvements in food consumption structure in China.

The structure of this paper is organized as follows. The theoretical background and hypotheses are discussed in Section 2. Sample data and measures are explained in Section 3. Empirical results are presented and discussed in Section 4. Conclusions and policy implications are presented in Section 5.

## 2. Theories and Hypotheses

Consumer knowledge refers to stored information or general background knowledge used to identify products in consumers’ memory [9], such as product properties, users’ experiences, etc. Knowledge focuses on consumers’ degree of familiarity with and level of expertise in products, as well as professional opinions about the products [10]. Studies have shown that knowledge affects consumers’ preferences for new products. For example, Suárez-Cáceres et al. [11] found that consumers’ knowledge significantly affected their attitudes and WTP for aquaponic products in Spain and Latin America. Particularly, consumers were more likely to pay a premium for products from aquaponics systems when the benefits of these products (i.e., the sustainability perspective) were highlighted [12,13]. Furthermore, some studies show that subjective knowledge has a stronger predictive effect on consumer purchase-related behavior than objective knowledge [14]. House et al. [15] found that a higher level of subjective knowledge would significantly improve the willingness to accept genetically modified foods, while objective knowledge did not play a role. Donoghue et al. [16] showed that subjective knowledge played a predictive role in the willingness of South African consumers to pay a premium for Karoo lamb, but that objective knowledge did not.

Animal welfare is a state of complete mental and physical health in which farm animals are in harmony with their surrounding environment. It generally refers to everything necessary to maintain animal physiology, mental health, and normal growth, such as good feeding, good housing, good health, and appropriate behavior [17]. Animal-derived products with high welfare standards are more guaranteed in terms of both nutritional quality and safety [18], and consumers are demanding safer, healthier, and higher quality foods under the same conditions [19]. If animal welfare information is readily available to the public, particularly through mass media, consumers will learn more about animal welfare and understand more about animal welfare products. Importantly, consumers can seek and find useful knowledge in their daily lives and use it to make purchase decisions, which implies consumers’ different subjective purchase intentions and preferences [20,21]. It can be inferred that if consumers learn more about animal welfare knowledge, they will realize that animal welfare is beneficial, and their acceptance of and preference for animal-friendly products are likely to increase. On this basis, we propose a hypothesis:

**Hypothesis** **1** **(H1).** *Consumers’ animal welfare knowledge has a positive effect on their behavioral intentions*.

Cognition is the process in which consumers choose, organize and explain external information and convert it into internal information [22]. It can be further specified as consumers’ perceptions of specific products [23], that is, the better consumers’ understanding of animal welfare products, the higher the level of consumer awareness. Consumers use knowledge to convert objective information into subjective cognition in purchasing decisions, thus affecting consumer attitudes and behaviors [24]. However, changes in consumer attitudes or perceptions of a product may affect one’s consumption habits [25]. Vermeir and Verbeke [26] argued that a positive attitude towards sustainable products was closely related to purchase intentions. Part of what makes animal welfare an important issue in livestock husbandry is that people can recognize the impact of improved animal welfare on the public or consumer utility [27]. Heng et al. [28] found that consumers’ cognition of animal welfare would be increased by appropriate education, promoting consumption of animal welfare products. Wang et al. [2] analyzed consumers’ understanding of animal welfare and perception of food safety using a survey conducted in Jiangsu Province, China. They found that consumers were willing to pay a certain premium for animal welfare. Yan et al. [29] and Clark [30] found that education level had a positive and significant impact on people’s understanding and behavioral intentions regarding animal welfare, and those with higher knowledge levels would be more likely to choose animal welfare products. Accordingly, animal welfare knowledge affects consumers’ understanding, judgment, and evaluation of animal welfare products, and thus affects their own consumption preferences and actual consumption behaviors. We propose a hypothesis:

**Hypothesis** **2** **(H2).** *Product cognition has a mediating effect on the relationship between animal welfare knowledge and consumers’ behavioral intentions*.

Various brain structures process information, and our decision-making processes involve both reason and emotion [31]. These two systems communicate with each other, impacting behavior together [32,33]. Empathy is an ability to put oneself in the position of others and to understand or feel what others have experienced [34] and contains cognitive factors that make people think from the perspective of people in need [35]. According to ecological ethics, animal welfare originates from humans’ moral responsibility for animals, and its core assumption is that animals have equal moral status and rights with humans to some extent [36]. Treating animals kindly conforms to people’s ethical cognition, affects people’s emotions, and increases people’s utility [37]. If animal welfare products arouse consumers’ empathy, consumers’ purchasing desire may increase [38]. Cornish et al. [39] found that younger females with lower household income had a higher level of empathy for animals, which was related to the intention to buy animal welfare products.

Empathy focuses on people’s subjective feelings about others, the other side of which is rational feelings and emotions. Product cognition is a comprehensive understanding of products, including composition, function, usage, advantages and disadvantages, characteristics, market, consumer groups, etc. It is the standard to measure consumers’ awareness and understanding of brand connotation and value. It can be seen that product cognition is based on objective feelings and rational emotions. Usually, rational consumers tend to make purchase decisions according to their familiarity with products. However, the decision-making process cannot be described as exclusively rational and conscious, as it is affected by emotional and subjective elements [40]. If emotion is a key factor in consumers’ purchase decision-making process [41], product cognition and behavioral intentions may be different between people with strong empathy and weak empathy. If consumers belong to the weak empathy group, their intentions to purchase or recommend may depend more on product cognition. If consumers belong to the strong empathy group, their purchase intention may rely more on empathy itself rather than product cognition. Specifically, consumers with strong empathy for animals may consider whether animals are suffering rather than whether animal products are delicious or not. That is, the effect of consumers’ cognition of the products on their behavioral intentions may be adjusted by empathy. Therefore, we hypothesize:

**Hypothesis** **3** **(H3).** *Empathy negatively moderates the relationship between knowledge of animal welfare and consumers’ behavioral intentions via product cognition.*

**Figure 2:**
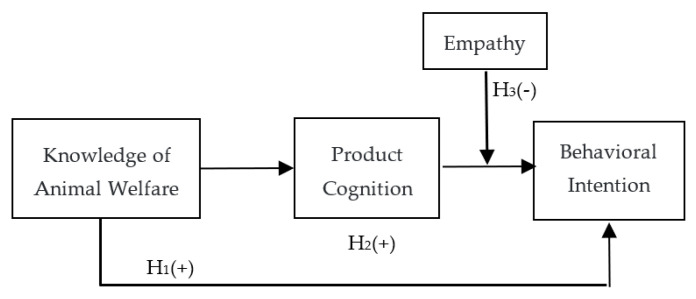
The conceptual framework of animal welfare knowledge, product cognition, empathy, and consumers’ behavioral intentions.

Figure 2 shows a conceptual model of this study. As indicated, product cognition mediates the relationship between knowledge of animal welfare and consumers’ behavioral intentions. In addition, empathy moderates the link between product cognition and consumers’ behavioral intentions. Thus, the indirect effect of knowledge of animal welfare on consumers’ behavioral intentions based on product cognition is strong when empathy is weak and weak when empathy is strong.

## 3. Data, Variables, and Methods

### 3.1. Data

The development of animal welfare is constrained by the level of regional economic and social development. In order to control costs, our survey was limited to major cities in Guangdong province. There were mainly two reasons for this. On the one hand, Guangdong province, neighboring two special administrative regions, Hong Kong, and Macao, is one of the provinces at the forefront of reform and opening up in China. Additionally, Guangzhou and Shenzhen, both located in Guangdong, are two of the top four cities, making Guangdong province a major province for foreign trade in China. On the other hand, Cantonese cuisine in Guangdong is one of the eight major cuisines in China. Cantonese people generally speak (or listen to) Cantonese, eat Cantonese cuisine, and have a good reputation for being "food experts". All of these facts help control the unobserved factors not controlled in the data, such as household food supply, regional tastes, diet, and other cultural factors.

Since we were interested in consumers’ knowledge of animal welfare and behavioral intentions toward animal-friendly products instead of societal opinions, our survey respondents were those who buy food for their families, eat meat products, and over the age of 16 years. As such, our survey may have avoided the bias problem of measuring consumers’ willingness to pay for private products with a sample including some consumers who buy the products for others.

Due to the coronavirus disease (COVID-19) outbreak worldwide, China has implemented strict epidemic prevention and control measures based on home isolation. Many Chinese people have had to transfer their daily studies, work, and lives to the Internet. For this reason, the questionnaires were uploaded onto Wenjuanxing (By recruiting and maintaining clientele, Wenjuanxing can sometimes offer consumers rewards for participating in surveys. The rewards offered to participants are points that they can collect and exchange for retail vouchers. Surveys are sent to participants randomly via email and online according to the sample requirements of the researchers. Participation in each investigation is voluntary. Researchers can contact and provide suppliers with questionnaires and pay fees in exchange for access to consumers who are prepared to participate in online surveys. The cost depends on the difficulty of the questionnaire, the duration of the survey, consumer characteristics and number of samples.), the first and largest domestic online questionnaire survey and test platform in China. Data were collected in two phases: a pilot survey and formal survey.

In February 2020, a pilot survey was conducted among 90 consumers mainly responsible for purchasing household food. In the pre-test, most respondents were not familiar with the concept of “animal welfare”. Searching for information is a key factor in the consumer decision-making process [42,43], and should be given enough attention in studies of consumer preferences [44]. Consequently, we clearly defined farm animal welfare and its products in the guide for the formal questionnaire. According to the feedback and suggestions of sample consumers, we revised the expressions in the questionnaire to make it simpler and easier to understand, removed the survey questions inconsistent with the local situation, and added some more valuable questions.

In March 2020, we conducted a formal survey through the Wenjuanxing platform and collected 1637 completed questionnaires in total. We treated completeness and quality of information as the screening criteria and eliminated invalid questionnaires that lacked crucial information or logic. Respondents with monotonous response behavior were also excluded because they may not have thoroughly read the questions or only completed the survey to obtain rewards. Finally, 1499 valid questionnaires with responses regarding demographics, meat consumption habits, knowledge of animal welfare, product cognition, and consumers’ behavioral intentions were obtained, with an effective response rate of 91.6%. All statistical analysis was carried out using Stata 16 (Stata Corp. 2019, created by StataCorp LLC, Texas, TX, USA).

### 3.2. Variables

#### 3.2.1. Dependent Variables

Purchase and recommendation are two common behaviors in consumers’ food decision-making. Following the practice of Weinrich et al. [45], we treated respondents’ willingness to purchase or recommend animal-friendly products as dependent variables to examine consumers’ behavioral intentions. Thus, the purchase intention variable measured one’s willingness to buy animal-friendly products and the recommend intention variable measured one’s willingness to promote animal-friendly products to friends. The above dependent variables were quantified by a 5-point scoring method, ranging from 1 for “absolutely not” buy or recommend to 5 for “always” buy or "absolutely" recommend. Table 1 shows that more than 80% of the respondents wanted to purchase and recommend farm animal welfare products.

#### 3.2.2. Core Independent Variables

At the beginning of the questionnaire, we asked respondents whether they knew about farm animal welfare. However, measuring animal welfare knowledge by simply asking respondents about their understanding of animal welfare is prone to bias. In order to make all respondents reach the same level before evaluating the dependent variables, we then provided a detailed description of the connotation of animal welfare. Respondents were told that animal welfare was a way of farming that met the basic natural needs of animals and kept animals in good living conditions, mainly including five freedoms for animals: freedom from hunger and thirst, freedom from discomfort, freedom from pain, injury and disease, freedom from fear and distress and freedom to express normal behavior [46].

In addition, respondents were informed that animal welfare products or animal-friendly products referred to products that meet animal welfare standards to varying degrees in the process of farm feeding, such as free-range chicken, eggs, pork, etc. According to the connotations of animal welfare and opinions that may easily be misunderstood by the public, we examined respondents’ cognition of animal welfare from four aspects: the ethical relationship between human beings and animals, weight between human welfare and animal welfare, opinions of animal health as well as the understanding of animal welfare cultural basis.

Table 2 presents items for evaluating consumers’ animal welfare knowledge in our questionnaire and the descriptive statistics of respondents’ answers to the questions. It can be seen that respondents had a high level of awareness of animal health and animal welfare culture, with correctness ratings of up to 88.99% and 88.59%, respectively. Unlike animal rights, animal welfare advocates the humane use of animals against any form of animal abuse rather than equating animals with humans. In this regard, 33.16% of respondents correctly understood this relationship, and 83.92% of respondents believed that animal welfare should not be considered before human welfare was guaranteed. It shows that most Chinese consumers tend to put people’s interests first when considering the relationship between humans and animals.

We further assigned values based on respondents’ answers to questions about animal welfare knowledge. Respondents who answered correctly would receive 1 point for one question, otherwise, 0, and thus, respondents’ knowledge of animal welfare was obtained. In order to observe the changes in respondents’ knowledge before and after providing animal welfare information, we also assigned values for respondents’ understanding of animal welfare before the survey, ranging from 0 for "do not know animal welfare at all" to 4 for "know animal welfare very well". Table 3 shows that 50.63 % of the respondents did not know about animal welfare at all, and 28.75% had heard of but did not know about animal welfare before receiving animal welfare information. This corresponded to 40.56% of respondents who obtained a full score of 4 points, and 41.96% who obtained 3 points after receiving animal welfare information. Although most respondents did not know about animal welfare at first, their cognitive level of animal welfare was significantly improved after a brief animal welfare literacy review during the survey. This indicates that Chinese consumers have good cognitive ability regarding animal welfare.

#### 3.2.3. Mediating Variables

Product cognition is the result of consumers internalizing objective animal welfare information about the product into subjective cognition, which in turn reflects consumers’ understanding of the quality of animal-friendly products. Animal welfare is related to attributes such as health [47], delicacy [7,48]), safety [49,50]), ethics [51,52]) and environmental friendliness [53,54,55]). Thus, we measured consumers’ cognition of animal welfare products from the five aspects mentioned above. The expression of the items was adapted from Carnovale et al. [56]. Each item was measured on a 5-point Likert scale, from “1” as “very disagree” to “5” as “very agree”. To avoid sequential effects, all items of the same scale were displayed at random in the survey. Internal consistency was measured using Cronbach’s α coefficient. Table 4 demonstrates that the reliability coefficient of each item was greater than 0.75, and total reliability was over 0.84, which was in line with the standard of a good reliability coefficient (above 0.6). The KMO value was close to 1 (0.82), the significance level of LR test results was less than 0.001 (chi-square value 3161.32). The reliability and validity were shown to be good.

#### 3.2.4. Moderating Variable

We measured empathy for animals by seeking respondents’ comments on the statement "I feel uncomfortable every time I see animals being abused or suffering" [57]. According to the survey results, the proportion of respondents reporting “very disagree”, “relatively disagree”, “general”, “relatively agree” and “very agree" was 3.94%, 3.67%, 17.95%, 46.43% and 35.62%, respectively. It can be seen that the respondents had a high level of empathy. In order to analyze the relationship between empathy and other variables more effectively, we merged the lowest three categories (“very disagree”, “relatively disagree” and “general”) into the level of “low”, making the distribution of categories that made up the moderating variable more balanced. Accordingly, empathy was defined on three levels: low, medium and high, corresponding to “disagree or generally agree”, “relatively agree" and "very agree”.

#### 3.2.5. Control Variables

Following previous research, we treated four kinds of demographic variables as controls. They included personal characteristics such as gender, age, years of education, household registration, family characteristics such as household income, number of people dining together, dining together with a child under 18, dining together with an elderly person over 60, and behavioral variables related to animal contact such as whether to raise pets, engaged in animal-related occupations, heard of animal welfare before. Additionally, the variable indicating whether the city of the respondent was a first-tier city was also included to control unobserved factors that were not clearly controlled in the data, such as regional economic development levels, household food supply, etc. Table 5 reports definitions and descriptive statistics of all variables used in the analyses.

### 3.3. Estimation Methods

Following the practices of Wykes et al. [58] and Preacher et al. [59], the proposed moderated mediation model can be tested by the stepwise method. In terms of the mediating effect test, the first step is to test whether the influence of independent variables on the dependent variable is significant, the second is to test whether the influence of independent variables on the mediating variable is significant, and the third is to test whether the influence of independent variables on the dependent variable is significantly reduced or even disappeared after controlling the mediating variable. The moderating effect is tested by constructing the interactive term between the independent variable and the moderating variable, and the moderating effect is judged by observing the significance level of the interaction. According to the theoretical hypothesis of this paper, the following moderated mediation model can be constructed:

(1) $$BI_{i}=\alpha_{10}+\alpha_{11}K_{i}+\alpha_{12}X+\varepsilon_{1}$$

(2) $$Awp_{i}=\alpha_{20}+\alpha_{21}K_{i}+\alpha_{22}X+\varepsilon_{2}$$

(3) $$BI_{i}=\alpha_{30}+\alpha_{31}K_{i}+\alpha_{32}Awp_{i}+\alpha_{33}X+\varepsilon_{3}$$

(4) $$BI_{i}=\alpha_{40}+\alpha_{41}K_{i}+\alpha_{42}Awp_{i}+\alpha_{43}Emp_{i}+\alpha_{44}Awp_{i}\times Emp_{i}+\alpha_{45}X+\varepsilon_{4}$$

In Equations (1)–(4): $BI_{i}$ represents consumers’ behavioral intentions, $K_{i}$ represents animal welfare knowledge, $Awp_{i}$ represents cognition of animal welfare products, $Emp_{i}$ represents animal empathy, and X represents a series of control variables mentioned above; $\alpha_{10}$, $\alpha_{20}$, $\alpha_{30}$, $\alpha_{40}$ are the corresponding constant terms; $\varepsilon_{1}$, $\varepsilon_{2}$, $\varepsilon_{3}$, $\varepsilon_{4}$ are the corresponding random error terms, which are assumed to be normal distribution; the subscript *i* denotes the *i*th respondent. Coefficient $\alpha_{11}$ in Equation (1) denotes the total effect of animal welfare knowledge on consumers’ behavioral intentions; coefficient $\alpha_{21}$ in Equation (2) denotes the impact of animal welfare knowledge on product cognition; coefficients $\alpha_{31}$, $\alpha_{41}$ in Equations (3) and (4) denote the direct effect of animal welfare knowledge on consumers’ behavioral intentions, and coefficients $\alpha_{32}$, $\alpha_{42}\;$ denote the direct effect of product cognition on consumers’ behavioral intentions. $\alpha_{21}\times \alpha_{32},\;$. which can be obtained by bringing Equation (2) into Equation (3), showing the indirect effect of animal welfare knowledge on consumers’ behavioral intentions, namely the impact of animal welfare knowledge on consumers’ behavioral intentions through product cognition. Coefficient $\alpha_{43}$ in Equation (4) represents the direct effect of the moderating variable on consumers’ behavioral intentions. $\alpha_{42}+\alpha_{44}Emp_{i}$ means the mediated effect of product cognition moderated by $Emp_{i}$ on consumers’ behavioral intentions.

## 4. Empirical Results and Analysis

### 4.1. Total Effect and Robustness Test

Table 6 reports the regression results of Equation (1). The OLS estimation results in columns (1) and (2) show that animal welfare knowledge has a significant positive impact on consumers’ behavioral intentions. Increasing animal welfare knowledge is conducive to improving consumers’ purchase and recommend intentions. In terms of control variables, women are more likely to recommend animal welfare products to others, which is consistent with the fact that women are more willing to share life experiences with others; young people in first-tier cities with better family economic conditions are more likely to accept animal welfare products; respondents with pet experiences prefer animal welfare products to those without pet experiences, and those that never heard of animal welfare are more willing to buy and recommend animal welfare products.

Some previous studies have shown that statistical results share a very similar significance level between ordinal and cardinal numbers [60]; the ordered Probit model is employed to estimate the impact of consumer welfare knowledge on consumers’ behavioral intentions. The ordered Probit model regression results in columns (3) and (4) of Table 6 are entirely consistent with the OLS regression results in the significance level and symbolic direction.

### 4.2. Mediating Effect of Product Cognition and Robustness Test

Considering that animal welfare knowledge has a significant effect on consumers’ behavioral intentions, we further explore product cognition’s mediating role. The stepwise regression method was used to estimate Equations (1)–(3), respectively, and robust standard errors were obtained. The estimation results are shown in Table 7. Column (1) and column (4), respectively, indicate the total effect of animal welfare knowledge on consumers’ purchase intentions and recommend intentions. Column (2) shows that animal welfare knowledge can significantly improve the levels of product cognition. Column (3) shows the direct effect of animal welfare knowledge and product cognition on consumers’ purchase intentions. The estimated coefficient of animal welfare knowledge variable was 0.077, which was significant at the 1% statistical level, indicating that the direct effect of animal welfare knowledge on purchase intention was 7.7%. The direct effect of product cognition on purchase intention was 0.263, significant at the 1% statistical level. When multiplying it with the animal welfare knowledge coefficient in column (2), we obtained the indirect effect of animal welfare knowledge on purchase intention (0.026), accounting for 25.24% of the total effect, which means that the mediating effect of animal welfare knowledge on purchase intention was about 25% by improving product cognition. Similarly, according to columns (2), (4), and (5) in Table 7, the mediating effect of animal welfare knowledge on recommend intention by increasing product cognition was about 21%.

Significance of indirect effects was estimated by the Sobel test and the bootstrap test. The Sobel test statistic *Z* for the purchase intention model was 4.113, and for the recommend intention model, 4.199. The associated *p*-values were both significant at the level of 1%, indicating significant mediation. Following the bootstrapping method of Preacher and Hayes [61] (setting 1000 iterations), results of the bootstrap test are shown in Table 8. The confidence intervals of both indirect and direct effects after bias correction did not include 0, indicating that there was indeed a transmission mechanism from animal welfare knowledge to purchase or recommend intention through improving product cognition. Both tests demonstrated that the stepwise regression method has a high degree of robustness for estimating the mediation effect.

### 4.3. Moderating Effect of Empathy and Robustness Test

To investigate the moderating role of empathy in the link between product cognition and consumers’ behavioral intentions, we constructed an interactive term between empathy and product cognition. Equation (4) was estimated using the OLS approach. The estimation results are shown in Table 9. The regression coefficient of the interactive term in column (2) was negative and significant at the level of 10%, showing that empathy may negatively regulate the positive impact of product cognition on purchase intention and recommend intention.

As mentioned above, the classification of the empathy variable may have an impact on the results. To check the robustness of the results and provide further insight into the relationship, all samples were divided into a strong empathy group and weak empathy group according to the mean of empathy. Subsequently, categorical analysis was conducted to compare the performance of the two groups (strong empathy vs. week empathy). The regression results in Table 9 show that the correlation coefficient of product cognition on purchase intention decreased from 0.320 in column (2) to 0.153 in column (3), which was significant at the level of 1%, indicating that the positive impact of product cognition on purchase intention decreases when empathy gains strength.

**Figure 3:**
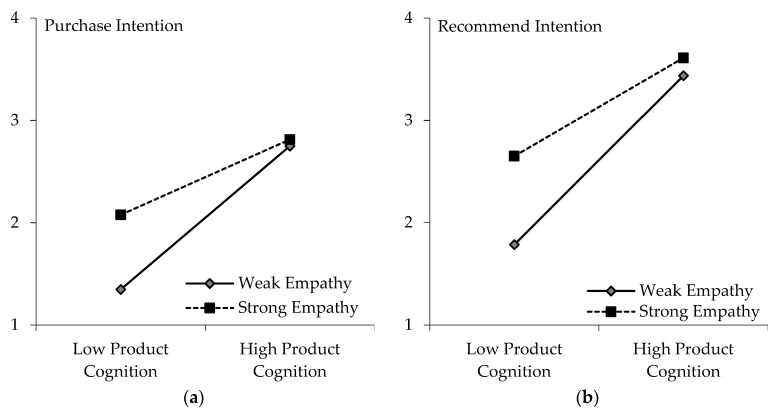
Effect of Empathy on Product Cognition Affecting Consumers’ Behavioral Intentions. (**a**) Purchase Intention, (**b**) Recommend Intention.

Figure 3 presents a more intuitive comparison of the regression results. It demonstrates that the purchase intention of the strong empathy group was stronger than that of the weak empathy group, which conformed to the expectation that empathy helps to promote animal welfare product consumption. The negative moderating effect of empathy was mainly manifested in the slope for the strong empathy group, which was smaller than that of the weak empathy group; that is, the impact of consumers’ product cognition on purchase intention in the strong empathy group was smaller than that in the weak empathy group, which was also in line with our expectations. The reason may be that empathy may be an important factor in motivating consumers to buy animal welfare products.

Animal welfare products are considered to possess both quality and ethical attributes. The strong purchase intention of the strong empathy group may be mainly due to their strong empathy for animals. In this regard, consumers’ purchase intentions would only be improved slightly even when the quality attributes of animal welfare products are identified. It is worth noting that animal welfare knowledge here did not have a significant impact on purchase intention for the strong empathy group. From another perspective, this may explain that consumers’ behavioral intentions regarding animal welfare products in the strong empathy group mainly depended on their animal ethics. For consumers with weak empathy, their purchase intentions for animal welfare products may be mainly due to quality attributes such as taste, health, quality, safety or other considerations. Improving their cognitive level of animal welfare products may be conducive for them to accept animal welfare products.

According to the results in columns (5) and (6) in Table 9, empathy moderates the effect of product cognition on recommend intention, similarly to the moderating effect of empathy on product cognition affecting purchase intention. Figure 3 also intuitively demonstrates that people’s recommend intentions for animal welfare products were stronger than their purchase intentions.

### 4.4. Further Discussions

This study investigated the relationship between animal welfare knowledge and consumers’ behavioral intentions with respect to animal welfare products with the mediating role of product cognition and the moderating role of empathy. Consumers’ perceptions and demands for farm animal welfare products determine the market outlook for animal husbandry producers to improve farm animal welfare and reflect public opinions and demands for the government to formulate laws, regulations and policies related to farm animal welfare.

This study found several interesting results. Animal welfare knowledge has a positive impact on consumers’ behavioral intentions, and increasing animal welfare knowledge may help improve consumers’ intentions to buy or promote animal welfare products. Our results were consistent with the findings in developing countries that consumers with positive attitudes towards animal welfare are willing to pay more for animal-friendly products [62,63,64]. Carnovale et al. [56] found that consumers with higher levels of animal welfare knowledge had higher purchase intentions for animal welfare products in China. In the past, it was generally believed that animal welfare was a matter for developed countries. However, the findings of this study show that that is not entirely the case. As a largest developing country, China has become an affluent society in a general sense since 2020, and people’s living standards have improved significantly. The demand for healthier, safer, higher-quality meat that meets their ethical requirements has been increasing. Therefore, a key issue going forward will be how to guide the public to fully understand animal welfare through publicity and promotional activities.

Furthermore, product cognition has a mediating effect on the link between animal welfare knowledge and consumers’ behavioral intentions. This finding indicates that increasing animal welfare knowledge helps to increase consumers’ product cognition. That is, consumers may link animal welfare knowledge with product cognition and make their purchase decisions. When acquiring knowledge about animal welfare, consumers may perceive and evaluate products with animal welfare attributes as of high quality because animal welfare products are generally known as high-end products with higher quality in nutrition, health, safety and taste than traditional products [5,18,65]. Hence, consumers may purchase more animal welfare products. Our findings support those of Jiang et al. [66], who suggested that positive animal welfare information made participants feel satisfied, healthy and happy, and consumers with higher product consumption showed a higher level of approval for animal welfare products. Consumers who often buy animal welfare products have more animal welfare knowledge and better product cognition. In addition to promoting animal welfare, it is necessary to achieve reasonable market segmentation and provide a more differentiated system for those who are interested in animal welfare, want higher standards of animal-derived products, and consider animal welfare in the search for information [67,68].

Finally, empathy plays a moderating role in the indirect effect of animal welfare knowledge on consumers’ behavioral intentions via product cognition. This finding suggests that consumers with strong empathy may integrate their concern for animals into their purchase decisions. The motive of perceptual consumption, or a type of moral consumption, may reduce the impact of product cognition on purchase or recommend intentions. Consumers with weak empathy are less affected by animal ethics. Instead, they mainly use animal welfare knowledge available to make purchase decisions by rationally evaluating product quality. Overall, consumers with strong empathy are more likely to purchase animal welfare products than consumers with weak empathy under the same conditions, possibly due to the dual influence of product cognition and animal ethics. The moderating effect of empathy on product cognition affecting consumers’ behavioral intentions confirms the ecological ethics of harmonious development between human and animals, and the existence of moral purchase behavior of consumers [69,70]. According to non-anthropocentrism, animals as the subject of life can feel pain and enjoy happiness. Freeing animals from unnecessary pain has become one of the motivations for consumers to consume animal welfare products. Therefore, promoting farm animal welfare not only fits the ethical perception of consumers but also has a functional impact on improving food safety [71]. However, consumers have to accept a higher price for the improvement of animal welfare, which to some extent inhibits consumer demand. Still, it is a fact that consumers can obtain additional benefits, such as health, deliciousness, safety, and even moral sentiment based on ecological ethics during animal welfare product consumption.

## 5. Conclusions and Implications

China is the most populated country in the world. Until now, people did not know much about animal welfare. Improving the level of animal welfare knowledge will help to cultivate market demand for farm animal welfare products and promote the high-quality development of animal husbandry. Based on the survey data of 1499 food consumers in Guangdong Province, China, this paper revealed the influence and orientation of animal welfare knowledge on consumers’ behavioral intentions by introducing product cognition and empathy.

The results from the moderated mediation model make three points. Firstly, the level of animal welfare knowledge has a significant positive impact on consumers’ behavioral intentions. Secondly, product cognition significantly increases consumers’ behavioral intentions and plays an intermediary role in the impact of animal welfare knowledge on consumers’ behavioral intentions. Thirdly, empathy has a significant positive effect on consumers’ behavioral intentions, and plays a negative moderating role through the indirect effect of animal welfare knowledge on consumers’ behavioral intentions via product cognition.

There are several policy implications to be drawn from the above findings. First, China needs to create a good institutional environment for animal welfare development in a planned way. In any case, improving animal welfare levels and product safety is an inevitable trend. Animal welfare will be an important attribute of high-end food in the future and an important factor restricting the high-quality development of animal husbandry. As farm animal welfare products are typical trust products, the formation of an effective market for these products requires that the government or third-party institutions establish regulatory measures to ensure the quality of farm animal welfare products. It is necessary for China to gradually establish and improve regulatory measures for the animal products market, strengthen the formulation and evaluation of animal welfare standards, and guide the meat production chain to improve its facilities, equipment and management, so as to meet the requirements of more sensitive markets.

Second, universal education pertaining to animal science is especially important. A growing interest in animal welfare can be attributed to urbanization, social education and economic development, as well as the influence of media and civil society organizations. Improving the level of animal welfare knowledge and awareness of animal welfare products helps to solve the problem of information asymmetry between consumers and producers. The Chinese government and related institutions can strengthen education in ecological ethics and animal welfare ideology by means of media, organizations, education and training in order to provide consumers with more information that they can use in the decision-making process.

Third, the precise positioning of people with high consumption tendencies is required. With China’s economy and society entering a transition period, people’s eating habits and food consumption patterns have diversified. Consumers are no longer limited to the issue of nutritional intake in the selection of animal-derived food, but have begun to pay more attention to taste, safety, health, and even ethical and environmental requirements. Consumers are the end receivers of animal-derived products, and their needs are dominant factors significantly affecting the development of farm animal welfare. If governments and enterprises perceive changes in consumer needs and that trend over time, they may find more new growth points and tap into new business opportunities. Enterprise marketing personnel can communicate with different customer groups according to the animal welfare characteristics of meat products to better meet the needs of customers and improve market share.

The present study has some limitations that deserve comment. Firstly, the selection of research samples was limited by social constraints, research costs and other practical factors. In the future, the number of samples and the coverage of respondents need to be expanded to further test the stability and universality of our findings. Secondly, this paper measured consumers’ behavior by behavioral intentions, and there was still a gap between intention and behavior. Therefore, future research on animal welfare product consumption can focus on consumers’ actual purchase behavior regarding animal welfare-related attribute products. Finally, this study controlled many factors such as personal and family characteristics, animal contact experience, level of city, etc. to explore the mechanism of animal welfare knowledge and product cognition affecting consumers’ behavioral intentions. The study was limited to cross-sectional data, and longitudinal design could better clarify consumers’ behavioral intentions and their influencing factors.

## Figures and Tables

**Table 1:** Consumers’ behavioral intentions regarding farm animal welfare products.

Purchase Intention			Recommend Intention		
Options	Number of People	Ratio	Layer	Number of People	Ratio
Absolutely not	55	3.67%	Absolutely not	20	1.33%
Rarely	135	9.01%	Rarely	151	10.07%
Sometimes	879	58.64%	Possibly	670	44.70%
Often	368	24.55%	Probably	509	33.96%
Always	62	4.14%	Absolutely	149	9.94%

**Table 2:** Answers to questions related to animal welfare knowledge.

Items	Correct	Wrong	Do Not Know
Animal welfare is completely equating animals with people.	33.16%	54.30%	12.54%
People’s welfare has not been achieved yet, so there is no need to consider animal welfare.	6.74%	83.92%	9.34%
Animal welfare considers both the “physical” and “mental” health of the animal	88.99%	3.87%	7.14%
Animal welfare conforms to people’s modern ecological and ethical requirements for animals.	88.59%	4.14%	7.27%

**Table 3:** Respondents’ mastery of animal welfare knowledge.

Types	0	1	2	3	4	Mean Difference
Self-statement	50.63%	28.75%	14.88%	4.67%	1.07%	−2.390 ***
Knowledge test	1.67%	3.94%	11.87%	41.96%	40.56%	

*** is statistically significant at 1% level.

**Table 4:** Cognition of farm animal welfare products.

Items	Mean	Standard Deviation	Reliability (Cronbach’s α)	Total Reliability
Meat from friendly-treated animals is healthier.	3.953	0.971	0.785	0.842
Meat from friendly-treated animals tastes better.	3.610	1.043	0.817	
Meat from friendly-treated animals is safer.	3.966	0.956	0.797	
It is more ethical to eat animal products with better welfare.	3.660	1.073	0.840	
Eating animal products with better welfare is better for the environment.	3.783	1.014	0.812	

**Table 5:** Definition and descriptive statistics of each variable.

Variables	Definition and Assigned Values	Mean	Standard Deviation
**Dependent variables**			
Purchase intention	Willingness to buy animal welfare products: Absolutely not = 1, Rarely = 2, Sometimes = 3, Often = 4, Always = 5	3.165	0.788
Recommend intention	Willingness to recommend animal welfare products: Absolutely not = 1, Rarely = 2, Possibly = 3, Probably = 4, Absolutely = 5	3.411	0.850
**Independent variables and control variables**			
AW knowledge	Scores of animal welfare knowledge test	3.158	0.899
Product cognition	Mean scores of five items for the respondent’s attitude towards animal welfare products	3.795	0.793
Empathy	Feelings every time a respondent sees animals being abused or suffering: Low = 1, Medium = 2, High = 3	2.177	0.710
Gender	Male = 1, Female = 0	0.361	0.480
Age	Age of the respondent	32.04	9.976
Dining scale	Number of people eating together in a family	3.853	1.463
Child	Whether there is a child under 18 years old dining together: Yes = 1, No = 0	1.518	0.500
Elderly	Whether there is an elderly person over 60 years old dining together: Yes = 1, No = 0	1.616	0.487
Income	Average household income per month, <6000 yuan = 1, 6000–12,000 yuan = 2, 12,000–18,000 yuan = 3, 18,000–24,000 yuan = 4, 24,000–30,000 yuan = 5, >30,000 yuan = 6	2.842	1.466
Education	Assigned values according to different educational levels: Primary school = 6, Middle school = 9, High school (Technical secondary or higher vocational school) = 12, Junior college = 14, Undergraduate = 16, Graduate or above = 19	15.32	2.440
Urban	Urban resident: Yes = 1, No = 0	0.849	0.359
Pet experience	Having experience of raising pets: Yes = 1, No = 0	0.229	0.421
Animal-related work	Engaged in animal-related occupations: Yes = 1, No = 0	0.049	0.217
Ever heard of AW	Heard of animal welfare before the survey: Yes = 1, No = 0	0.794	0.405
First-tier city	Living in Guangzhou or Shenzhen: Yes = 1, No = 0	0.726	0.446

**Table 6:** Total effects of animal welfare knowledge on consumer’s behavioral intention.

Variable	(1)	(2)	(3)	(4)
	OLS	OLS	Ordered Probit	Ordered Probit
	Purchase Intention	Recommend Intention	Purchase Intention	Recommend Intention
AW knowledge	0.103 ***	0.151 ***	0.144 ***	0.195 ***
	(0.025)	(0.025)	(0.035)	(0.032)
Gender	−0.027	−0.106 **	−0.039	−0.138 **
	(0.042)	(0.046)	(0.061)	(0.059)
Age	0.010 ***	0.007 ***	0.016 ***	0.009 ***
	(0.003)	(0.003)	(0.004)	(0.003)
Education	−0.003	−0.010	−0.005	−0.014
	(0.010)	(0.011)	(0.014)	(0.014)
Urban	0.035	0.044	0.065	0.059
	(0.058)	(0.061)	(0.083)	(0.079)
Income	0.048 ***	0.038 **	0.068 ***	0.050 **
	(0.016)	(0.017)	(0.023)	(0.022)
Dinning scale	0.016	0.011	0.025	0.014
	(0.019)	(0.019)	(0.026)	(0.025)
Child	−0.014	−0.048	−0.024	−0.063
	(0.048)	(0.051)	(0.068)	(0.066)
Elderly	0.060	−0.027	0.089	−0.037
	(0.048)	(0.051)	(0.068)	(0.067)
Pet experience	0.106 **	0.133 **	0.158 **	0.175 ***
	(0.048)	(0.052)	(0.069)	(0.068)
Animal-related work	0.111	0.112	0.165	0.149
	(0.101)	(0.114)	(0.143)	(0.147)
Ever heard of AW	−0.101 **	−0.099 **	−0.145 **	−0.128 **
	(0.041)	(0.044)	(0.059)	(0.057)
First-tier city	0.096 **	0.066	0.147 **	0.085
	(0.048)	(0.050)	(0.068)	(0.065)
Constant	2.216 ***	2.800 ***		
	(0.287)	(0.309)		
Observation	1499	1499	1499	1499
R-squared/Pseudo R2	0.055	0.057	0.026	0.024
Wald chi2			81.86	91.43
Log pseudo-likelihood			−1646.082	−1821.981

*** and ** are statistically significant at 1% and 5% levels, respectively. Numbers in parentheses are robust standard errors.

**Table 7:** Mediating effects of product cognition.

Variables	(1)	(2)	(3)	(4)	(5)
	Purchase Intention	Product Cognition	Purchase Intention	Recommend Intention	Recommend Intention
AW Knowledge	0.103 ***	0.098 ***	0.077 ***	0.151 ***	0.119 ***
	(0.025)	(0.023)	(0.024)	(0.025)	(0.024)
Product Cognition			0.263 ***		0.330 ***
			(0.035)		(0.036)
Control variables	controlled	controlled	controlled	controlled	controlled
Constant	2.216 ***	3.069 ***	1.408 ***	2.800 ***	1.788 ***
	(0.287)	(0.281)	(0.299)	(0.309)	(0.306)
Observation	1499	1499	1499	1499	1499
R-squared	0.055	0.046	0.121	0.057	0.147

*** is statistically significant at 1% level. Numbers in parentheses are robust standard errors. Due to space limitations, only brief results are represented.

**Table 8:** Bootstrap test results for mediating effect.

Dependent Variables	Bootstrap Test	Coefficient	Deviation	Standard Deviation	95% C.I.	Bias-Corrected C.I.
Purchase intention	indirect effect	0.027	−0.000	0.007	[0.015,0.042]	[0.016,0.045]
	direct effect	0.077	−0.000	0.024	[0.032,0.124]	[0.033,0.124]
Recommend intention	indirect effect	0.034	−0.000	0.008	[0.019,0.051]	[0.019,0.052]
	direct effect	0.117	−0.000	0.025	[0.070,0.167]	[0.069,0.166]

**Table 9:** Moderating effects of empathy.

Variables	(1)	(2)	(3)	(4)	(5)	(6)
	Purchase Intention			Recommend Intention		
	All Sample	Weak Empathy Group	Strong Empathy Group	All Sample	Weak Empathy Group	Strong Empathy Group
AW Knowledge	0.062 **	0.088 ***	0.022	0.097 ***	0.122 ***	0.082
	(0.024)	(0.027)	(0.051)	(0.023)	(0.025)	(0.055)
Product Cognition	0.433 ***	0.320 ***	0.153 ***	0.499 ***	0.372 ***	0.232 ***
	(0.108)	(0.041)	(0.059)	(0.110)	(0.039)	(0.062)
Empathy	0.448 **			0.519 ***		
	(0.190)			(0.195)		
Product Cognition × Empathy	−0.083 *			−0.086 *		
	(0.048)			(0.049)		
Control Variables	controlled	controlled	controlled	controlled	controlled	controlled
Constant	0.551	0.892 ***	2.560 ***	0.854 *	1.473 ***	2.635 ***
	(0.503)	(0.345)	(0.571)	(0.515)	(0.347)	(0.595)
Observation	1499	965	534	1499	965	534
R-squared	0.139	0.166	0.066	0.177	0.195	0.091

***, ** and * are statistically significant at 1%, 5% and 10% levels, respectively. Numbers in parentheses are robust standard errors. Due to space limitations, only brief results are represented.

## Data Availability

All study data used for analysis are available upon request.
